# Metformin Improves the Prognosis of Adult Mice with Sepsis-Associated Encephalopathy Better than That of Aged Mice

**DOI:** 10.1155/2022/3218452

**Published:** 2022-05-04

**Authors:** Gaofei Song, Huoyan Liang, Heng Song, Xianfei Ding, Dong Wang, Xiaojuan Zhang, Tongwen Sun

**Affiliations:** ^1^General ICU, The First Affiliated Hospital of Zhengzhou University, Henan Key Laboratory of Critical Care Medicine, Zhengzhou Key Laboratory of Sepsis, Henan Engineering Research Center for Critical Care Medicine, Zhengzhou, China; ^2^Academy of Medical Sciences, Zhengzhou University, Zhengzhou, China

## Abstract

Sepsis-associated encephalopathy (SAE) is often associated with increased ICU occupancy and hospital mortality and poor long-term outcomes, with currently no specific treatment. Pathophysiological mechanisms of SAE are complex and may involve activation of microglia, multiple intracranial inflammatory factors, and inflammatory pathways. We hypothesized that metformin may have an effect on microglia, which affects the prognosis of SAE. In this study, metformin treatment of mice with SAE induced by lipopolysaccharide (LPS) reduced the expression of microglia protein and related inflammatory factors. Poor prognosis of SAE is related to increased expression of tumor necrosis factor-*α* (TNF-*α*) and interleukin-1 beta (IL-1*β*) in brain tissues. Levels of inflammatory cytokines produced by LPS-induced SAE mouse microglia were significantly increased compared with those in the sham group. In addition, ionized calcium-binding adapter molecule 1 (Iba-1) was significantly reduced in metformin-treated SAE mice compared with untreated SAE mice, suggesting that metformin can reduce microgliosis and inhibit central nervous system inflammation, thereby improving patient outcomes. In conclusion, our results stipulate that metformin inhibits inflammation through the adenosine 5′-monophosphate (AMP-) activated protein kinase pathway by inhibiting nuclear factor kappa beta (NF-*κ*B). Metformin can partially reverse the severe prognosis caused by sepsis by blocking microglial proliferation and inhibiting the production of inflammatory factors.

## 1. Introduction

Sepsis is defined as life-threatening organ dysfunction caused by a dysregulated host response to infection [1, 2] and most commonly results in multiple organ dysfunction syndromes in severely affected patients [3]. In sepsis, the central nervous system (CNS) is considered to be one of the first systems involved [4], and the mortality rate increases with the severity of SAE, rising up to 70% [5]. The main clinical outcome is poor prognosis and high mortality rates. SAE is characterized by diffuse brain dysfunction and cognitive impairment as a result of systemic inflammation caused by infection, with clinical manifestations ranging from delirium to coma. However, no clinical or laboratory evidence exist of direct brain infection, brain anatomical abnormalities, cerebral hemorrhage, or cerebral infarction [6]. The pathophysiology of SAE is complex and multifactorial, combining interwoven processes, which lead to numerous functional alterations and disorders, such as neuroinflammation, oxidative stress, reduced brain metabolism, and impaired integrity of the blood-brain barrier (BBB) [7]. In addition, peripheral cytokine storm 8 leads to the production of intracranial inflammatory mediators and plays a key role in the pathogenesis of SAE, mainly manifested as activation of microglia, leukocyte infiltration, and neuronal degeneration. Meanwhile, the permeability of the BBB increases [9], and the infiltration of peripheral inflammatory mediators into the CNS further enhances BBB permeability and promotes the production of inflammatory mediators.

Microglia are important participants in developing the CNS-related disorders, inflammation, and almost all neuropathological conditions, such as stroke, tumors, degenerative diseases, brain injury, and infection, accounting for approximately 5–12% of the total number of intracranial cells [10, 11]. These cells have the morphological and functional ability to adapt to the ever-changing microenvironment around them. Microglial activation in response to stimulation is widely believed to promote neurotoxin clearance and maintain intracranial homeostasis [12, 13]. Iba-1 is considered to be a marker protein of microglia [14]. During normal development, microglia participate in the clearance of aging neurons through phagocytosis without causing an inflammatory response [15, 16]. Under pathological conditions such as SAE, microglia are rapidly activated to produce large amounts of nitric oxide, TNF-*α*, IL-6, IL-1, oxygen-free radicals, and various excitatory neurotransmitters. This expanded inflammatory cascade aggravates neuronal damage, thus damaging the state of consciousness and emotional states [17, 18]. Activated microglia can produce a variety of inflammatory factors, such as TNF-*α*, IL-1*β*, and NF-*κ*B [19, 20]. NF-*κ*B, as an inducible transcription factor, plays a central role in the regulation of inflammatory genes [21]. Lipopolysaccharide (LPS) can strongly stimulate the activation of microglia and is widely used in the study of neuroinflammation [22]. Since disordered activation of microglia during SAE may lead to exacerbation of existing brain damage, regulation of microglia appears to be a therapeutic option for SAE [23, 24]. Metformin has been found to reduce mortality in patients with sepsis significantly. It also protects the lung tissue from oxidative damage caused by sepsis [25]. In contrast to other classes of hypoglycemic drugs, metformin inhibits NF-*κ*B activation through the adenosine 5′-monophosphate-activated protein kinase (AMPK) pathway [26]. It more recently has also been shown to benefit nondiabetic patients by reducing the production of inflammatory agents and biomarkers of aging [27]. A close link between reduced AMPK activity and inflammation has been revealed in the studies of macrophages by Yang et al. [28]. Moreover, in 2013, Jiang et al. have found that metformin can reduce intracranial infarction area, cell apoptosis, and neurological deficits caused by focal cerebral ischemia through the AMPK autophagy pathway in cerebral ischemia diseases [29]. However, few studies have focused on the role of metformin in SAE. Therefore, we aimed to investigate the effect and potential role of metformin on LPS-induced inflammation during SAE.

## 2. Materials and Methods

This study conducted in accordance with the principles outlined in the National Institutes of Health (NIH) Guide for the Care and Use of Laboratory Animals, and Zhengzhou University Life Sciences Ethics Review Committee approved the experimental protocols.

### 2.1. Reagents

The antibody to TNF-*α*, IL1-beta, AMPK, and *β*-actin was obtained from proteintech (Wuhan, China) and Phospho-AMPK*α* (1 : 1000) get from Cell Signaling Technology (Wuhan, China). The research group purchased Uelris RT Mix with DNsae (All-in-one) reagent from UE (Suzhou, China) Company and Hieff®qPCR SYBR Green Master Mix (Low Rox Plus) reagent from Yessen Biotechnology (Shanghai, China) Co., Ltd.

### 2.2. Animals

In October 2019, 35 male C57BL/6 mice (4 weeks of age, 15-20 g) were purchased from the Beijing Charles River Experimental Animal Technology Center (Beijing, China) (5 of them were reserved to prevent various accidental deaths) and were raised under standard conditions for 1 year. This group of mice is called aged mice (13 months old). In October 2020, 30 male C57BL/6 mice (4 weeks of age, 15-20 g) were purchased from the Beijing Charles River Experimental Animal Technology Center (Beijing, China) again and were fed for 1 week under standard conditions. This group of mice is called adult mice. Two groups of mice (aged mice and adult mice) were randomly divided into three groups with 10 mice in each group: sham group, LPS group, and LPS + M group. LPS group and LPS + M group were intraperitoneally injected with LPS 1 mg·kg-1, and metformin 25 mg·kg-1 was intraperitoneally injected with LPS + M group 1 h later. The standard feeding conditions of mice were as follows: temperature: 18~22°C, observe the temperature every day, adjust the temperature if necessary. Relative humidity: 50~60%, daily estimate humidity with wet and dry bulb thermometer, take measures to adjust humidity if necessary. Noise: below 60 decibels. Lighting: 10~14 hours, the light is on at 8 : 00 every day, 18 : 00~20 : 00 lights. Ammonia: below 20 PPM. Ventilation: 8 ~20 times/hour. Airflow: 10~25 cm/min. Cage tools: generally, the pad material in the cage of mice is changed once a week, and the cage tools are washed and disinfected once a month. Drinking bottle: supply clean and pollution-free drinking water. Wash your drinking bottle every 3 days and add fresh, clean water. Boil once a month for disinfection. Feeding room: indoor disinfection with 0.1% geramine spray every month; peracetic acid was fumigated quarterly.

### 2.3. Murine Sepsis Score (MSS)

We assessed the severity of sepsis in each mouse according to MSS score. The mice in each group were scored from 8 a.m. to 10 a.m. every day, including appearance, respiratory rate and quality, presence of eye secretions, activity, and response to stimuli [30]. The higher the score, the more severe the sepsis. When the mouse score reached 21 points, the mice were judged dead. The mice were euthanized immediately, and the brain tissue was removed and stored in liquid nitrogen. Two trained students rated the MSS of the mice.

### 2.4. Neurobehavioral Score

After MSS score was given to confirm sepsis in mice, a neural score was then used to determine whether the mice had SAE. We scored each group of mice from 8 a.m. to 10 a.m. every day, including auricle reflex, corneal reflex, and escape response. Mice with no reflexes scored 0. One point was scored for the mice whose reflexes were weaker than usual. Mice with normal reflexes scored 2 points [31]. Two trained students rated the MSS of the mice.

### 2.5. Hematoxylin and Eosin (HE) and Immunofluorescence

We assessed mouse brain tissue previously stored in 4% paraformaldehyde using hematoxylin-eosin (HE) staining. After paraffin embedding, the brain was cut into 4 *μ*m sections and stained with HE. The histological changes of brain tissue were observed under a microscope. Immunofluorescence: after dewaxing and hydration, cells are given permeability, sealed endogenous peroxidase, antigen repair, exposure to antigenic determiners, sealed nonspecific proteins, incubation of primary and secondary antibodies, color rendering after SP reaction, etc. The expression of IBA-1 in the hippocampal CA1, CA3, and DG regions was detected to observe the number of microglia. Microglia were labeled with Cy3 red fluorescence.

### 2.6. Western Blot Analysis

Previously stored mouse brain tissue was removed from liquid nitrogen. After labeling, brain tissue lysates were prepared, and the protein concentration was determined by the BCA method with reference to the standard curve. The gel was prepared according to the method of polyacrylamide gel, and then the protein was transferred to nitrocellulose membrane by electrophoresis and electric transfer. The protein was sealed with skim milk powder in saline containing Tween (TBST) buffer for 2 hours. The primary antibody solution was prepared according to different antibody instructions. The nitrocellulose membrane was soaked in TBST and incubated in a 4° shaker for 12-16 hours. The nitrocellulose membrane was again incubated with the secondary antibody at room temperature for 2 hours and exposed. Specific bands were detected by chemiluminescence, and the ECL signal was digitally processed by Bandscan 5.0 software.

### 2.7. qPCR Analysis

Previously stored brain tissue is removed from liquid nitrogen. Trizol reagent was added after grinding, and the purified total RNA was obtained by two-phase separation, RNA precipitation, RNA cleaning, and precipitation of dissolved RNA after RNA drying in strict accordance with Trizol RNA extraction kit process. The concentration and purity of RNA were measured by spectrophotometer after absorption value. CDNA was prepared according to the instructions of the Uelris RT Mix with DNsae (all-in-one) reverse transcription kit. Hieff®qPCR SYBR Green Master Mix (Low Rox Plus) kit instructions are for amplification. PCR results were analyzed using the −*ΔΔ* 2^Ct^ method. Gene expression data are shown relative to the control, which was set at 100%. Gene expression levels were normalized to those of GAPDH. The primers used are shown in Table 1.

### 2.8. Statistical Analysis

We used the mean ± SEM to express the data and used the one way analysis of variance and Tukey's post hoc methods to estimate the differences among the three groups, and *p* < 0.05 was considered significant. The SPSS 21.0 software was used to analyse all results from this study.

## 3. Result

### 3.1. Metformin Improves the Prognosis of SAE Mice

**Figure 1:**
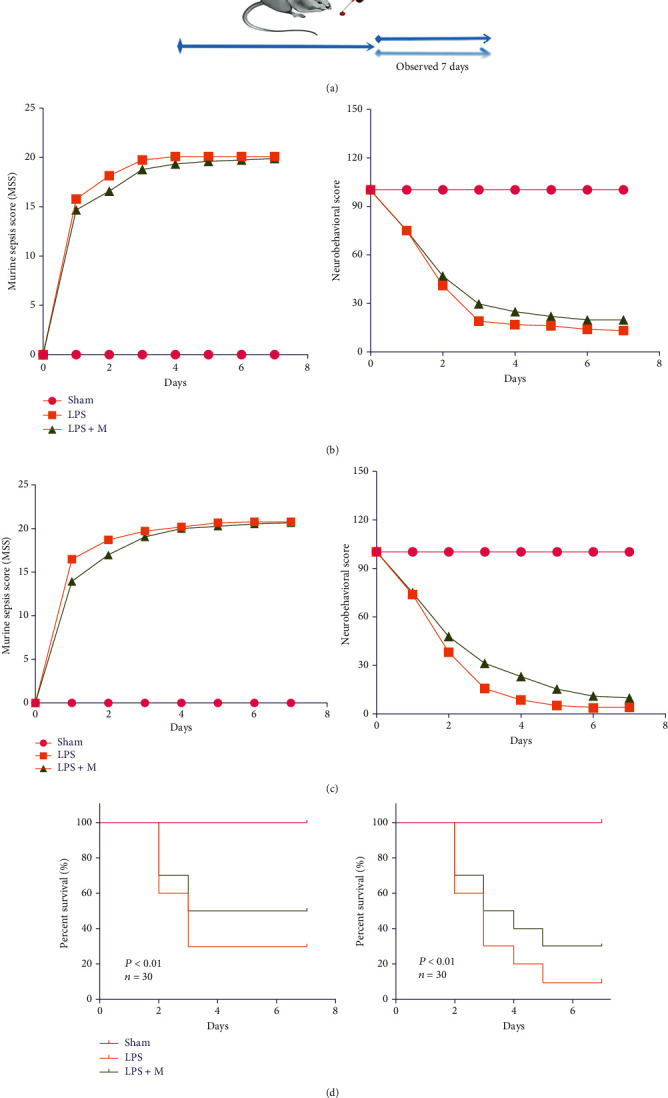
(a) Mouse model diagram. (b) MSS score and neurobehavioral score of dentate gyrus of adult mouse brain tissue. (c) MSS score and neurobehavioral score of dentate gyrus of aged mice brain tissue. (d) Survival curves of adult and aged mice. The mice were intraperitoneally injected with LPS, then, given metformin 1 hour later. The vital signs of the mice were observed for the following 7 days, and MSS score and neurobehavioral score were given every day. When THE MSS score reached 21, the death was determined, and the brain tissue was dissected for use. In the legend of the survival curve, the mortality rate of the aged mice after LPS administration was significantly higher than that of the adult group, while the effect of metformin treatment in the aged group was significantly lower than that in the adult group. LPS: lipopolysaccharide.

When the MSS score of experimental mice reached 21 points, the mice were actively euthanized, and their tissues were dissected and stored in liquid nitrogen. As shown in Figure 1(b), the MSS scores of the adult LPS group on days 2 and 3 were significantly higher than those of the sham group (*p* < 0.05). Compared with the LPS group, the LPS + M group showed decreased MSS scores at 2–3 days after sepsis induction. As shown in Figure 1(b), the MSS score in the LPS group was significantly higher than that in the sham group on days 2 and 3 after sepsis induction (*p* < 0.05), while the LPS + M group had decreased MSS scores, indicating that metformin can improve the prognosis of SAE mice. Neurobehavioral scores were used to further observe effects of LPS-induced sepsis in the brain. Scores of the adult and aged LPS groups on days 2 and 3 were significantly lower than those of the sham group. On days 4 and 5 after sepsis induction, the neurobehavioral scores of LPS + M group were increased compared with the LPS group (Figures 1(b) and 1(c)), indicating that metformin improved the neurobehavioral performance of mice with LPS-induced sepsis. The 7-day survival rate of the LPS sepsis-induced mouse model is shown in Figure 1(d). In the adult group, the 7-day mortality rates of the sham, LPS, and LPS + M groups were 0%, 70%, and 50%, respectively. The 7-day mortality rates in the old group were 0%, 90%, and 70%, respectively. The mortality rate in the LPS group was significantly higher than that in the sham group (*p* < 0.001) and LPS + M (*p* = 0.3613). In addition, compared with the adult LPS group, increased mortality (*p* < 0.001) was found in the aged LPS group, while there were no statistically significant difference between the aged and the adult LPS + M groups (Table 2).

### 3.2. Metformin Reduces the Infiltration of Microglia during SAE

**Figure 2:**
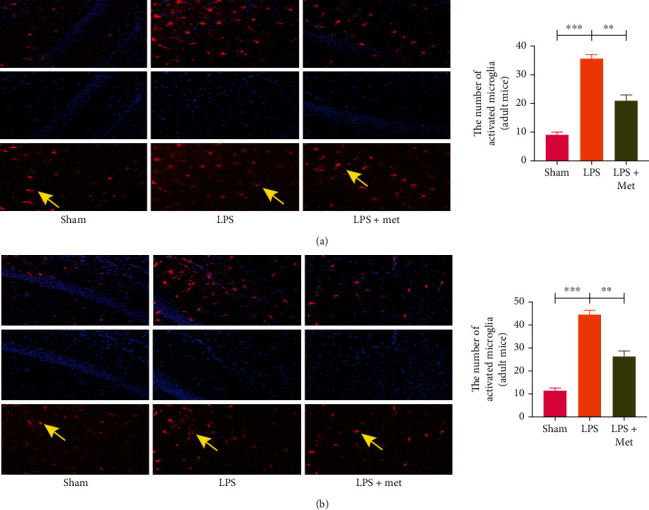
(a) Fluorescent staining of adult mouse microglia. (b) Fluorescent staining of aged mice microglia. As shown in figure (a) and (b), microglia were marked with Cy3 red. Activation of microglia in the metformin-treated group was significantly reduced compared with that in the LPS group. As shown by the arrow: activated microglia. In figure (a) and (b), they are shown from top to bottom: the above is the synthesis of the middle and lower figures, and the middle figure: the nuclei are dyed blue by DAPI method; below: microglia IBA-1, shown in red. ∗*p* < 0.05, ∗∗*p* < 0.01, ∗∗∗*p* < 0.001. *n* = 10 in each group.

**Figure 3:**
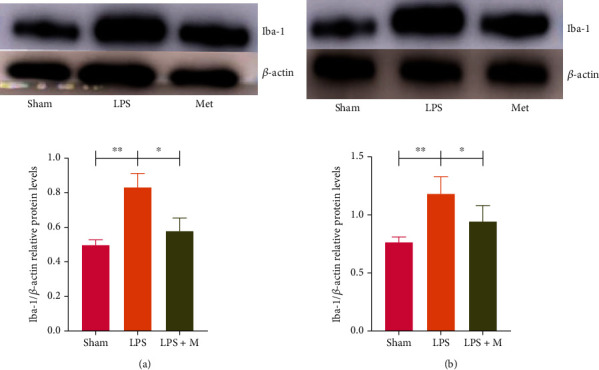
(a, b) Effect of metformin on the level of inflammatory factor protein in brain tissue of SAE-induced mice. In figures (a) and (b), the expression level of Iba-1 in LPS + M group was lower than that in LPS group, indicating that metformin could inhibit the activation of microglia. ∗*p* < 0.05, ∗∗*p* < 0.01, ∗∗∗*p* < 0.001. *n* = 10 in each group; Iba-1: ionized calcium-binding adapter molecule 1; LPS: lipopolysaccharide; LPS + M: lipopolysaccharide and metformin.

In the LPS-induced sepsis model treated with metformin, the immunofluorescence staining of microglia showed that the numbers of microglia in the adult and aged groups were significantly higher than those in the sham groups (*p* < 0.05). Microglia were significantly reduced in the LPS + M group compared with the LPS group (Figures 2 and 3) (*p* < 0.05). Western blots showed that the microglial marker Iba-1 was significantly increased in the LPS group (*p* all < 0.05), indicating that microglia were significantly activated in LPS-induced sepsis mice. However, in the LPS + M group, microglial Iba-1 protein expression was decreased compared with that in the LPS group (*p* all <0.05). These results suggest that metformin can inhibit microglial activation in mice with LPS-induced sepsis (Figures 2 and 3).

### 3.3. Metformin Improves Inflammatory Factor Infiltration into Brain Tissue of SAE Mice

**Figure 4:**
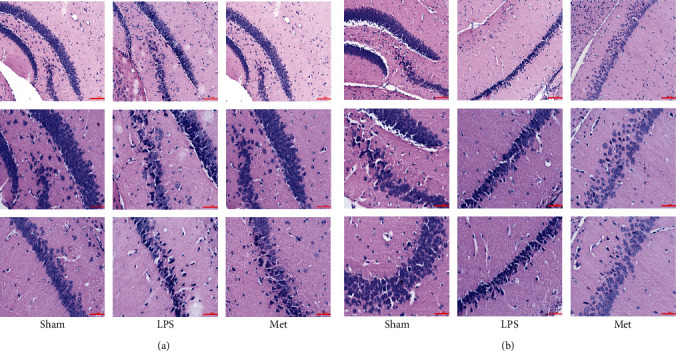
(a) HE staining of adult group, (b) HE staining of aged group. HE staining showed that inflammation and cell arrangement disorders were observed in both adult and aged mice after LPS injection, especially in dentate gyrus. In the metformin-treated group, the aged mice had more inflammatory cells than the adult mice.

**Figure 5:**
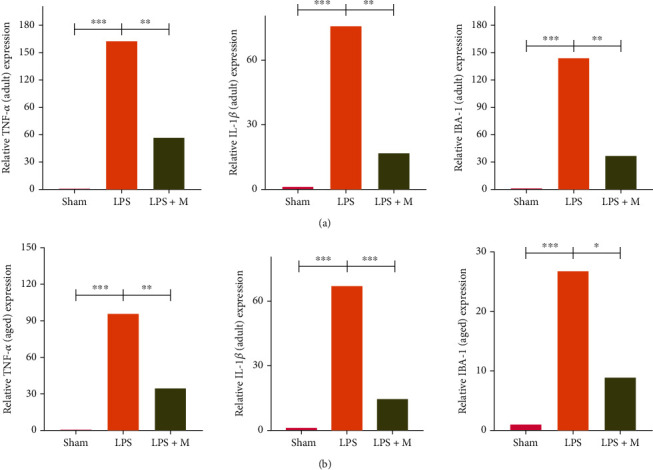
Effect of metformin on the expression of inflammatory factor gene in brain tissue of SAE-induced mice. The gene expression levels of inflammatory factors, TNF-*α*, IL-1*β*, and Iba-1 were detected by qPCR assay among groups (a, b). ^∗^*p* < 0.05, ^∗∗^*p* < 0.01, ^∗∗∗^*p*  < 0.001. *n* = 10 in each group. qPCR: quantitative polymerase chain reaction; TNF-*α*: tumor necrosis factor-*α*; IL-1*β*: interleukin-1*β*; LPS: lipopolysaccharide; LPS + M: lipopolysaccharide and metformin.

**Figure 6:**
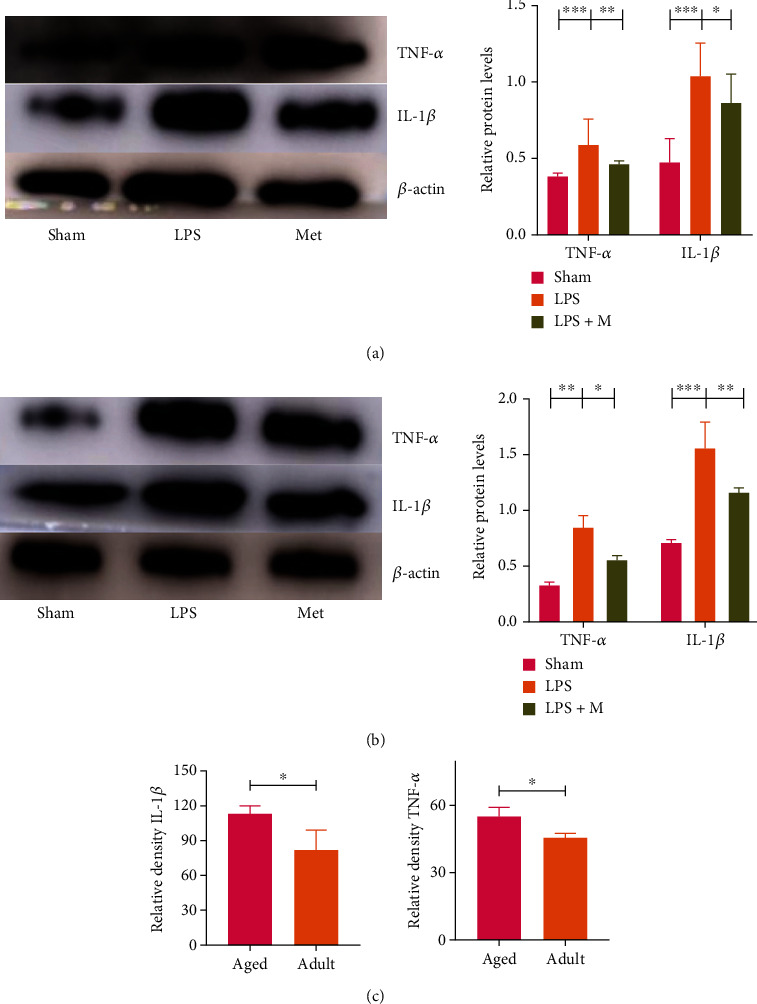
(a, b) Effects of metformin on TNF-*α* and IL-1*β* protein levels in LPS-induced SAE mice brain tissue. (c) Comparison of LPS-induced SAE mice treated with metformin between the adult group and the aged group LPS + M group. ^∗^*p*  < 0.05, ^∗∗^*p* < 0.01, ^∗∗∗^*p* < 0.001. *n* = 10 in each group. TNF-*α*: tumor necrosis factor-*α*; IL-1*β*: interleukin-1*β*; LPS: lipopolysaccharide; LPS + M: lipopolysaccharide and metformin.

In the LPS-induced SAE model of metformin, inflammatory cell infiltration, disorder of cell arrangement, and cell edema were observed in HE staining of brain tissue in the adult and aged LPS groups, while inflammatory cell infiltration and cell edema were decreased in the adult and aged LPS + M groups compared with their respective LPS groups (Figures 4(a) and 4(b)). Western blot and qPCR showed that TNF-*α* and IL-1*β* levels in the LPS group were significantly higher than those in the sham group, while these cytokines were higher than those in the LPS + M group (*p* all < 0.05). Thus, metformin significantly reduced inflammatory cytokines after SAE (Figures 5, 6(a), and 6(b)) (*p* all < 0.05).

### 3.4. The Adult SAE Group Was More Responsive to Metformin than the Aged Group

**Figure 7:**
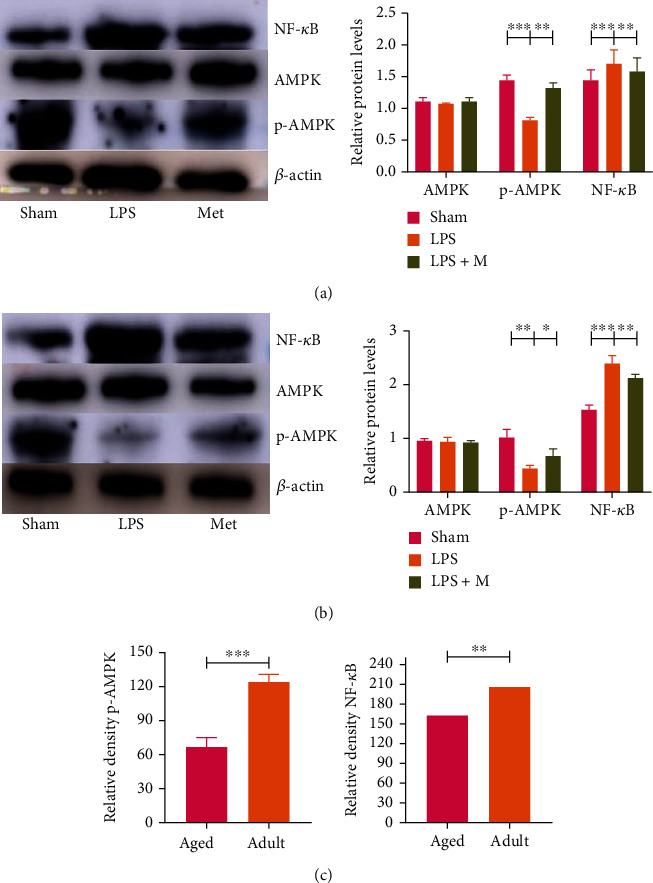
(a) and (b) show the effects of metformin on AMPK, p-AMPK, and NF-*κ*B protein levels in LPS-induced SAE mice brain tissues in adult and aged groups, respectively. (c) Comparison of LPS-induced SAE mice treated with metformin between the adult group and the aged group LPS + M group. ^∗^*p* < 0.05, ^∗∗^*p* < 0.01, ^∗∗∗^*p* < 0.001. *n* = 10 in each group. AMPL: adenosine 5′-monophosphate- (AMP-) activated protein kinase; p-AMPK: phosphorylation adenosine 5′-monophosphate- (AMP-) activated protein kinase; NF-*κ*B: nuclear factor kappa-B.

After metformin treatment, expression levels of inflammatory factors in the adult SAE group were significantly lower than those in the aged group (Figure 6(c)) (*p* all <0.05). After metformin treatment, p-AMPK and NF-*κ*B levels in the adult SAE group were significantly lower than those in the aged group (Figure 7(c)) (*p* all < 0.05). In conclusion, metformin reduced the inflammatory response in adult mice better than in aged mice.

### 3.5. Metformin Acts on LPS-Induced SAE through AMPK-NF-*κ*B Pathway

Metformin prevents inflammation by inhibiting the transcription factor NF-*κ*B through a pathway dependent on AMPK. We found that NF-*κ*B, TNF-*α*, and IL-1*β* protein expression levels were decreased in both adult and aged LPS + M groups compared with the LPS group (*p* all < 0.05), suggesting that metformin alleviates LPS-induced inflammatory response through the AMPK-NF-*κ*B pathway, thereby improving the prognosis of SAE (Figures 6(a), 6(b), 7(a), and 7(b)).

## 4. Discussion

The results of this study established that metformin reduced microglia and various inflammatory factors in mice with LPS-induced SAE and inhibited this inflammatory response by inhibiting nuclear factor NF-*κ*B in an AMPK-dependent pathway, thereby improving prognosis. To the best of our knowledge, we used aged mice as the LPS-induced SAE target for the first time, which was consistent with the prevalence of SAE in aged patients in clinical practice, and can better reflect the pathophysiological status of aged patients.

SAE has an unsatisfactory therapeutic outcome over the course of the disease, and many patients have a poor prognosis and reduced quality of life after treatment. SAE is characterized by an immune response to bacterial endotoxin in the absence of an obvious central infection [32]. People will inevitably be infected with viruses, bacteria, and fungi in their whole life. Due to the body's own immunity, not every time will lead to sepsis. When the body appears aging, major trauma, and other conditions, the overstrong immune response leads to the body's own organs becoming the target of inflammatory factors, affecting the central nervous system, we call it sepsis-associated encephalopathy [33]. SAE patients often have no obvious infection of the central nervous system, but neuroinflammation and oxidative stress responses are present in brain tissue. Patients can have obvious cognitive dysfunction, memory decline, fuzzy consciousness and coma, and other neurological symptoms [34]. Early in SAE, microglia in the nervous system are rapidly activated and secrete large amounts of cytokines, leading to inflammation of the blood-brain barrier and central nervous system [35]. The function of normal microglia in the central nervous system is to phagocytose and remove damaged nerve cells and tissue fragments, but the continuous inflammatory reaction abnormally enhances the function of microglia, leading to the active attack of normal neurons and nerve tissue in the central nervous system [36]. Therefore, we chose the LPS-induced experimental SAE model and carried out relevant experiments using this model. The LPS-induced SAE model induces the release of multiple inflammatory factors (TNF-*α* and IL-1*β*) from microglia, leading to neuronal damage and a severe prognosis [37]. Microglia are “specialized” immune cells of myeloid origin with the function of detecting and removing pathogens, damaged cells, and cell debris. Activated microglia-mediated neuroinflammation is associated with neuronal death and exacerbation of neurodegenerative disease [38]. Previous studies have demonstrated a correlation between microglia activation and SAE [39]. Overactivation of microglia may account for the poor prognosis of SAE disease [40]. Abnormally activated microglias mediate neuroinflammatory response by producing a variety of proinflammatory factors, which is an important pathological process in CNS diseases [41]. Reducing the activation of microglia, thereby reducing the production of inflammatory factors and subsequent neuronal damage, offers potential treatment options for neuroinflammatory diseases [42, 43]. IL-1*β* has been implicated in a variety of CNS diseases, most of which are associated with inflammatory processes [44]. Microglia play an increasingly important role in the treatment of SAE and may be a new therapeutic target [45, 46]. In our study, immunofluorescence showed that a large number of microglia were activated in the LPS group, while fewer microglia were activated in the LPS + M group (Figures 4(a) and 4(b)). These results showed that metformin reduced the number of microglia in mice with LPS-induced SAE and improved their prognosis.

To further investigate the possible effects of metformin on microglia during LPS-induced SAE, we examined expression levels of inflammatory cytokines at the protein and gene levels. Inhibition of NF-*κ*B signal transduction can reduce LPS-induced microglial inflammatory mediators, thereby reducing intracranial inflammation and improving prognosis [47, 48]. According to western blotting and qPCR results, metformin can inhibit LPS-induced TNF-*α*, IL-1*β*, and NF-*κ*B production. Excessive microglial production, which in turn produces excess pro-inflammatory factors, leads to neuronal damage [49, 50]. Amoani et al. found that high dose of metformin in peripheral blood circulation can inhibit TNF-*α* production and indirectly reduce inflammatory response [51]. Some studies have found that metformin can promote the conversion of microglia from the M1 (proinflammatory) type to the M2 (anti-inflammatory) type, which has been shown to protect nerve cells [52]. In the aged animals, Senescence-associated secretory proteins (SASP) can release many proinflammatory factors, such as IL-6 and IL-1*β*. These cytokines can cause insulin resistance, chronic inflammation, and an increased risk of cancer [53, 54]. Metformin indirectly alleviates inflammation by inhibiting NF-*κ*B production and reducing SASP secretion [55, 56]. The potential mechanisms whereby metformin reduces cardiovascular event incidence and all-cause mortality have been actively investigated. Metformin not only alleviates hyperglycemia and insulin resistance [57, 58] but also other cardiovascular risk factors, such as overweight or obesity, atherosclerosis, blood pressure, procoagulant state and thrombosis, and carotid intima thickening [59, 60]. In addition, metformin treatment reduced the numbers of pathogenic Th17 cells and increased the numbers of regulatory T cells, thereby limiting the inflammatory response [61, 62]. Some studies have shown that patients with sepsis treated with chronic metformin have a lower mortality rate than patients not treated with metformin [63]. Other studies have shown no differences in serum lactate clearance between patients with sepsis treated with chronic metformin and those not treated with metformin [64]. Although antibacterial effects of metformin are not fully understood, the underlying metabolic mechanisms of metformin are widely attributed to the activation of AMPK [65]. Toejing et al. found that metformin ameliorates endotoxemia and inflammation through the AMPK-NF-*κ*B pathway [31, 66]. Mammalian AMPK is generally thought of as a sensor for adenine nucleotides, activated in a state of low cellular energy. It restores energy balance by turning on catabolic pathways that produce ATP, while shutting down anabolic pathways and other ATP-consuming processes [67, 68]. During SAE, impaired intracranial hemodynamics result in reduced oxygen and energy supply to microglia and neurons, ultimately leading to AMPK activation. Jian et al. found that AMPK is also involved in inflammatory responses by regulating NF-*κ*B signaling [69]. Wang et al. reported that activation of AMPK downregulates NF-*κ*B pathway function [70, 71], with phosphorylated AMPK inhibiting NF-*κ*B activation and decreasing IL-1*β* and TNF-*α* production [72, 73]. These results suggest that AMPK/NF-*κ*B may play an important role in reducing the inflammatory response and improving prognosis during SAE [74]. In the peripheral system, metformin alleviates LPS-induced macrophage-induced acute lung injury through the AMPK pathway [75]. Microglia are considered to function as macrophages in the central nervous system, and our study demonstrates that metformin reduces SAE-induced inflammation by reducing microglia activation. In our experiment, the MSS and neurobehavioral scores were used to determine the occurrence and severity of sepsis. Western blotting and qPCR showed that the expression of IL-1*β* and TNF-*α* decreased in the LPS + M group. Metformin may improve the prognosis of mice by inhibiting the release of related inflammatory factors through the AMPK-NF-*κ*B pathway. Our results also showed that metformin treatment in the adult LPS group was more effective than in the aged LPS group.

The shortcoming of this paper is that we did not conduct further cell experiments to verify the inflammatory pathways. And we tried to mimic the human condition of geriatric sepsis in aged mice, but we gave metformin only once. If we inject metformin intraperitoneally for 14 consecutive days in aged mice, and then inject LPS intraperitoneally to create sepsis model, it may be more consistent with the human disease model and may better reflect the role of metformin in the treatment of sepsis.

## 5. Conclusion

Overall, our results suggest that metformin reduces microglia and various inflammatory factors in mice with LPS-induced SAE and may inhibit the inflammatory response by inhibiting NF-*κ*B in an AMPK-dependent pathway, thereby improving prognosis. The expression of inflammatory factors in adult mice was lower than that in aged mice, suggesting that metformin treatment in adult mice was more effective compared with older mice. It can be concluded that the protective potential of metformin is worth considering to reduce the inflammatory responses during SAE to improve prognosis.

## Figures and Tables

**Table 1:** Primers used for real-time PCR.

Genes	Sense primers (5′-3′)	Antisense primers (5′-3′)
Iba-1	ATTATGTCCTTGAAGCGAATGC	TCTCAAGATGGCAGATCTCTTG
NF-*κ*B	CTCAGAGCCAGCCCAGGCTT	CGCACTTGTAACGGAAACGC
TNF-*α*	GCATACAGGTCCTGGCATCT	TTCTTGCTGGTCTTGCCATT
GAPDH	GATGGTGAAGGTCGGTGTG	GAGGTCAATGAAGGGGTCG

Note. PCR: polymerase chain reaction.

**Table 2:** Mortality of aged groups and adult groups among groups.

Groups	Mortality (number of death/total)	*p* values
LPS (adult)	70% (7/10)	0.0062^a^
LPS (aged)	90% (9/10)	
LPS + M (adult)	50% (5/10)	0.3613^b^
LPS + M (aged)	70% (7/10)	

Note. LPS: lipopolysaccharide; LPS + M: lipopolysaccharide + metformin. ^a^Comparison with LPS (aged). ^b^Comparison with LPS + M (aged). The mortality at 7 days in the LPS (aged) group was significantly higher than that in the LPS (adult) (*p* < 0.001). The mortality at 7 days in the LPS + M (aged) group was not significantly higher than that in the LPS + M (adult) (*p* = 0.3613).

## Data Availability

The data used to support the findings of this study are included within the article.
